# Advances in rapid detection technologies for zoonotic diseases: a one health-oriented review

**DOI:** 10.3389/fcimb.2026.1791165

**Published:** 2026-07-07

**Authors:** Jidong Zhao, Xiang Hou, Gang Chang

**Affiliations:** Shaanxi Key Laboratory of Qinling Ecological Security, Shaanxi Institute of Zoology, Xi’an, China

**Keywords:** isothermal amplification, one health, point-of-care testing, rapid detection, wildlife reservoir surveillance, zoonotic diseases

## Abstract

Zoonotic diseases account for the majority of emerging infectious threats and pose unique diagnostic challenges because their surveillance and detection must bridge human, animal, and environmental health sectors. Unlike conventional infectious disease diagnostics, rapid detection of zoonotic pathogens often requires adaptation to cross-species transmission dynamics, heterogeneous sample matrices derived from wildlife, livestock, humans, and environmental sources, as well as field-based surveillance in resource-limited settings. In this review, we summarize recent advances in rapid detection technologies for zoonotic pathogens, including immunological assays, molecular diagnostics, isothermal amplification platforms, biosensors, and microfluidic systems. More importantly, we examine these technologies through a zoonosis-specific perspective by evaluating their suitability for major surveillance scenarios such as wildlife reservoir monitoring, farm-level screening, outbreak-site emergency detection, and laboratory confirmation. We further discuss how the One Health framework reshapes technical demands for pathogen detection, particularly in relation to sample complexity, portability, multiplexing, biosafety, and real-time data integration. Finally, we highlight current bottlenecks and future directions toward integrated, intelligent, and field-deployable diagnostic systems. This review provides a scenario-oriented and One Health-based reference for selecting and developing rapid detection tools for zoonotic disease surveillance and control.

## 1 Introduction

Zoonotic diseases constitute one of the most important sources of emerging infectious threats worldwide. More than 60% of emerging infectious diseases are estimated to originate from animal reservoirs, and many recent outbreaks—including COVID-19, avian influenza, brucellosis, and hemorrhagic fever of varied origin—have demonstrated how rapidly pathogens can move across animal, human, and environmental interfaces. Such spillover processes are shaped by intensifying livestock production, increased human encroachment into wildlife habitats, global trade, climate-associated ecological change, and expanding human mobility (Ellwanger and Chies, 2021; Keesing and Ostfeld, 2021). As a result, zoonotic disease surveillance is no longer limited to clinical diagnosis in humans (Keesing and Ostfeld, 2021; Bernstein et al., 2022), but increasingly depends on integrated monitoring across wildlife, domestic animals, food systems, and environmental sources (Jones et al., 2013; Keesing and Ostfeld, 2021; Zhang et al., 2024b).

Rapid detection technologies are central to this effort because early pathogen identification directly affects outbreak response speed, containment efficiency, and downstream prevention strategies. However, the diagnostic requirements of zoonotic diseases differ in important ways from those of conventional human infectious diseases. Zoonotic pathogens often circulate across multiple host species, requiring diagnostic systems that can be applied to diverse biological matrices (Nichols and Geddes, 2021; Chen and Meng, 2025). Besides, surveillance frequently involves such heterogeneous samples, including blood, serum, feces, oral and nasal swabs, animal tissues, milk, food products, wastewater, and environmental samples, all of which may contain inhibitors or low pathogen abundance (Maggi et al., 2025). Many zoonotic surveillance activities occur outside centralized laboratories, such as on farms, in slaughterhouses, wildlife monitoring stations, border checkpoints, and remote field sites, thereby increasing demands for portability, robustness, biosafety, and ease of operation (Sharan et al., 2023). Overall, under the One Health framework, diagnostic technologies are increasingly expected not only to confirm infection, but also to support cross-sector surveillance, early warning, and coordinated risk assessment.

**Figure 1:**
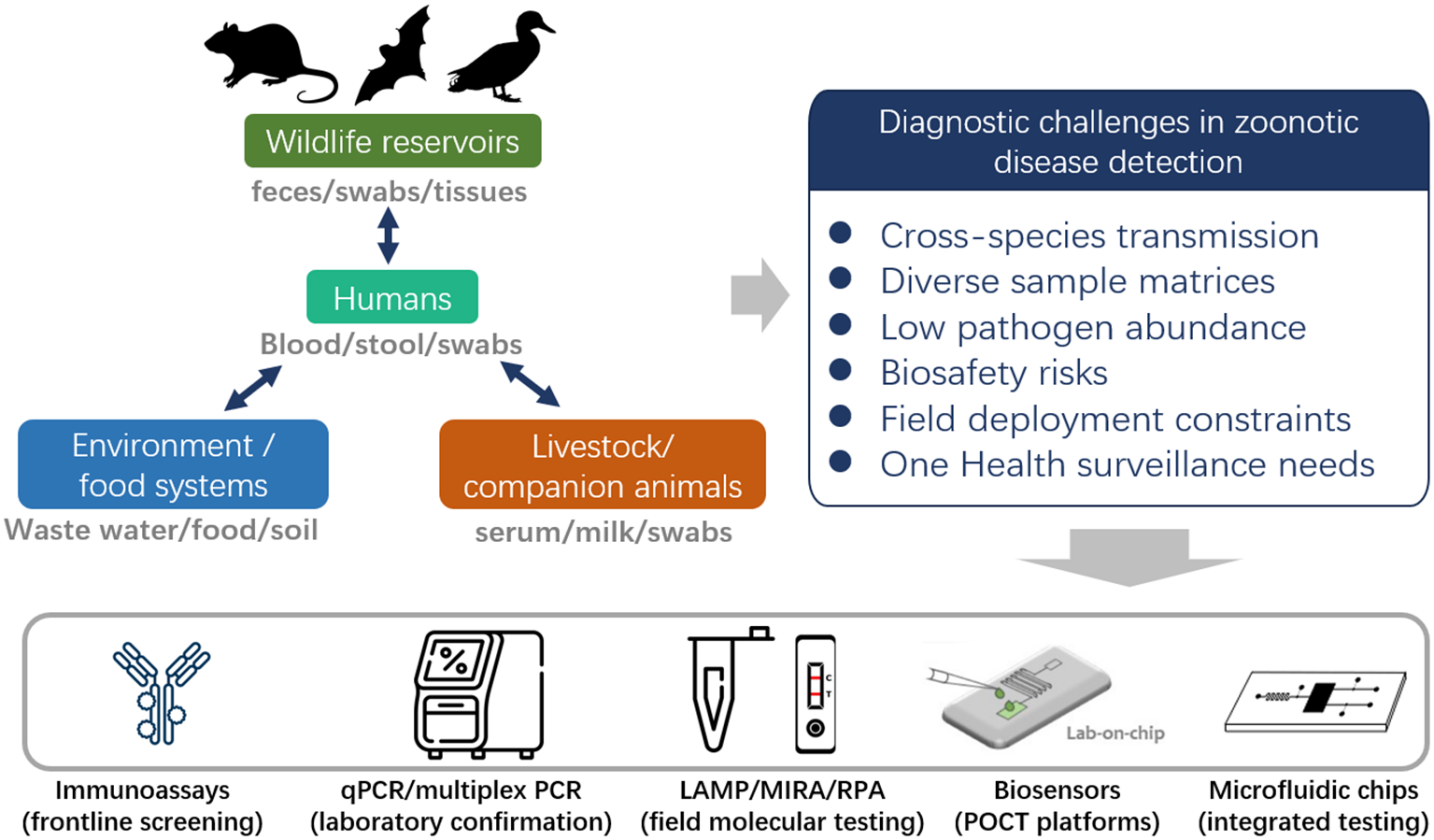
One Health interfaces, diagnostic challenges, and rapid detection strategies for zoonotic diseases. Zoonotic disease surveillance covers interconnected interfaces of wildlife reservoirs, humans, livestock and companion animals, as well as environmental and food systems, involving diverse biological samples. Under the One Health framework, these considerations call for scenario-adapted rapid detection technologies. The representative strategies below constitute a complementary diagnostic system for zoonotic disease surveillance and control.

Existing reviews have summarized rapid diagnostic technologies for infectious diseases in general, often focusing on analytical sensitivity, specificity, and platform innovation (Sharan et al., 2023; Olatunji et al., 2024; Kramme et al., 2025). However, fewer reviews have systematically examined how these technologies should be selected, adapted, and integrated for zoonotic disease surveillance under real-world One Health scenarios. In this review, we therefore analyze major rapid detection platforms from a zoonosis-oriented perspective. We first outline the diagnostic challenges that are specific to zoonotic diseases, including cross-species transmission, heterogeneous sample types, wildlife reservoir surveillance, and decentralized field deployment. We then summarize the principles, strengths, and limitations of major rapid detection technologies, including immunological assays, qPCR, multiplex PCR, rapid nucleic acid extraction methods, isothermal amplification systems, biosensors, and microfluidic chips. Importantly, we further compare their adaptability across major zoonotic surveillance scenarios, such as farm-level screening, wildlife monitoring, emergency outbreak response, and laboratory confirmation. Finally, we discuss current bottlenecks and future directions toward integrated, multiplexed, intelligent, and field-deployable diagnostic systems for zoonotic disease control. To better illustrate these interconnected challenges and the overall surveillance framework, a One Health-based conceptual overview is presented (**Figure 1**). This framework highlights the interactions among wildlife reservoirs, humans, livestock or companion animals, and environmental systems, and summarizes the key diagnostic challenges that distinguish zoonotic disease detection from conventional infectious disease diagnostics, thereby providing a conceptual basis for the scenario-driven selection and application of rapid detection technologies.

## 2 Diagnostic challenges specific to zoonotic diseases

### 2.1 Cross-species transmission creates unique diagnostic demands

Most conventional infectious diseases only circulate within single host groups, while zoonoses are defined by obligate or facultative cross-species transmission among humans, livestock and wild animals. This biological trait raises distinct diagnostic requirements that differ from those for single-host pathogens (Kruse et al., 2004; Rahman et al., 2020). Pathogens may persist silently in wild animal reservoirs, proliferate in domestic animals before spilling over to humans, or spread backward from humans to animals via reverse zoonosis (Messenger et al., 2014; Al Noman et al., 2024).

Differences in pathogen loads, shedding rules, tissue tropism and clinical manifestations across different host species further complicate diagnostic system design. Detection methods effective for human clinical samples often fail to fit wildlife monitoring and veterinary screening scenarios. For this reason, rapid detection techniques for zoonoses need to be assessed not only for basic analytical performance, but also for their adaptability to diverse hosts and different transmission phases.

### 2.2 Diverse sample matrices undermine detection consistency

Zoonotic disease monitoring under the One Health system involves biological samples sourced from humans, animals and environmental media, forming highly diversified detection objects. Apart from routine human clinical specimens, field surveillance commonly adopts animal serum, whole blood, feces, swabs, tissue homogenates, dairy products, carcass tissues, wastewater, soil samples, vector carriers and food matrices (Sharan et al., 2023; Ali et al., 2026).

These samples differ greatly in pathogen content, background microflora, inhibitory substances and biosafety risks, making sample pre-treatment a core influencing factor for successful detection (Adedokun et al., 2024). Optimized zoonotic detection tools need to adapt to complex matrices, reduce reliance on high-purity nucleic acid extraction, and keep steady detection efficiency when applied to various field-collected specimens.

### 2.3 Field surveillance: portability requirements and practical deployment barriers

Many zoonotic pathogens are persistently maintained in wildlife reservoirs such as bats, rodents, birds, and wild ungulates (Mkomwa et al., 2025). Traditional laboratory-based detection approaches cannot be widely applied in such regions. Field monitoring urgently requires lightweight, sturdy and user-friendly rapid detection platforms that support closed-tube operation and low-pollution testing (AsSudani et al., 2026; Liu et al., 2026). Meanwhile, field sampling and testing face high biosafety risks due to potential exposure to unknown pathogens. On-site detection technologies should balance detection sensitivity, simple operation and biological safety management.

Harsh field environments such as high humidity, extreme temperature and dust pollution also accelerate nucleic acid degradation and lower detection accuracy. Wildlife sampling also brings logistical difficulties and safety risks, forcing most specimens to be delivered to central laboratories for testing. This process delays diagnosis, increases monitoring costs and slows down epidemic response speed, severely restricting early pathogen screening and real-time risk assessment in high-risk spillover zones.

### 2.4 Obstacles in zoonotic surveillance data integration

Zoonotic monitoring generates massive heterogeneous data from medical institutions, animal epidemic prevention stations, wild animal investigation projects, vector surveys and environmental testing platforms (Chame et al., 2019). Related data cover clinical case records, pathogen genome sequences, serological test results, ecological survey data, animal mobility information and environmental pathogen monitoring data (Johnson et al., 2018; Nguyen-Tien et al., 2025). Different industries and administrative departments adopt inconsistent sampling strategies, detection methods, professional terminologies and data management norms. This leads to huge differences in data formatting, metadata annotation, calibration standards and result judgment criteria across sectors. Divergences in sampling frequency, laboratory detection level and statistical standards further lower data compatibility and comparability.

Fragmented monitoring systems hinder cross-department data sharing, real-time epidemic tracing, pathogen spillover tracking and integrated risk assessment, and delay the early warning and joint prevention of emerging zoonoses (Oltean et al., 2025). Solving such data fusion difficulties has become a key task of One Health collaborative research, aiming to strengthen interdisciplinary cooperation and build integrated monitoring mechanisms.

### 2.5 Bottlenecks restricting simultaneous multi-pathogen detection

Wild and domestic animal hosts often carry various viruses, bacteria, fungi and parasites, leading to prevalent co-infections in natural ecosystems. Pathogen interactions change disease progression patterns, obscure clinical symptoms and interfere with epidemic traceability work (Ellwanger and Chies, 2021; Li et al., 2025b). Most conventional detection methods are designed for single-target identification, requiring separate sample processing and repeated testing. Such modes consume massive manpower and financial resources, slow down emergency response procedures and fail to meet large-scale field monitoring demands in under-resourced areas (Osei Sekyere, 2025). In multi-pathogen parallel detection systems, primer competition, non-specific binding and signal interference easily cause false positive results and miss low-abundance pathogens (Calchi et al., 2024; Sakkos et al., 2026). Moreover, frequent genetic mutations reduce the stability of existing detection targets.

Although next-generation sequencing, multi-channel qPCR and CRISPR-based methods support simultaneous screening of multiple pathogens, their large-scale promotion is limited by high costs, complicated data analysis processes, incomplete reference gene databases and unclear standards to distinguish real infection signals from environmental pollution interference (Li et al., 2023; Lei and Xu, 2024).

Future detection optimization can combine high-sensitivity biosensors, portable sequencing equipment and intelligent data analysis technology. Low-cost and field-adaptable multi-pathogen testing tools are essential to improve early warning capability and cross-species transmission monitoring efficiency. Combined analysis with epidemiological and ecological data can also clarify pathogen evolution rules and zoonotic spillover mechanisms. With increasing human-wildlife contact and accelerating ecological changes, integrated multi-pathogen monitoring under One Health has become increasingly essential.

### 2.6 Economic and resource constraints

Zoonoses are highly prevalent in low- and middle-income regions and remote rural areas with frequent cross-contact among humans, livestock, wild animals and disease vectors (Okeke and Ihekweazu, 2021). These regions generally lack complete medical and laboratory infrastructure, stable power supply and sound cold-chain systems, which greatly restrict the popularization of advanced detection technology (Luis et al., 2013; Han et al., 2016).

High-end molecular detection means including real-time PCR and high-throughput sequencing require huge capital investment, professional laboratories and skilled operators, making them inaccessible for grassroots routine monitoring. Expensive equipment, costly imported reagents, daily maintenance fees and cold-chain transportation costs also affect long-term stable operation, and reagent shortage during epidemics will directly block emergency prevention work (Okeke and Ihekweazu, 2021; Boro and Stoll, 2022). Regional resource gaps further widen the monitoring disparity: developed regions have formed complete integrated surveillance systems, while high-risk spillover areas lack basic molecular testing conditions, resulting in delayed pathogen discovery, underreported cases and weakened global epidemic early warning networks (Sharan et al., 2023; Sun et al., 2024).

Isothermal amplification, CRISPR detection and portable sequencing technologies fit the usage demands of low-resource regions, yet they still need further optimization in stability, simplified sample treatment, multi-target detection ability and unified operating specifications to match One Health monitoring requirements (Boro and Stoll, 2022; Sharan et al., 2023; Sun et al., 2024). Long-term progress relies on international academic cooperation, local reagent production, technology transfer and regional laboratory construction, to realize equitable popularization of affordable detection tools and sustain long-term monitoring at the human-animal-environment interface.

### 2.7 Balanced optimization of detection sensitivity and specificity

Balancing sensitivity and specificity remains a key technical hurdle in zoonotic pathogen identification, especially when testing complex biological and environmental samples. Specimens collected from wild animals, livestock, vector organisms and environmental carriers usually contain low-concentration pathogens, fragmented nucleic acids and diverse interfering substances. Hemoglobin, polysaccharides, humic acid and host endogenous proteins can inhibit nucleic acid amplification and interfere with result judgment (Meisner et al., 2023; Milićević et al., 2024).

In early infection or latent infection stages, pathogen content is often lower than the threshold of conventional testing methods, easily generating false negative results and hiding potential epidemic risks (Gao and Fu, 2025). Excessively improving detection sensitivity through super-sensitive amplification or broad-spectrum target screening will trigger non-specific amplification, cross-reaction with genetically similar microbes and external pollution interference, thus lowering specificity and producing false positive data (Petrie et al., 2025; Cohen et al., 2026). This defect is more obvious in multi-host ecosystems with abundant homologous pathogens and common co-infections.

Differences in animal species, sample types and variable pathogen mutation patterns also make it difficult to unify testing protocols and guarantee consistent detection effects in diverse monitoring scenarios (Oliveira Roster et al., 2024). Researchers need to optimize primer and probe sequences, adjust amplification procedures, upgrade sample pre-treatment methods and set scientific detection thresholds, so as to stabilize dual indicators of sensitivity and specificity for practical field application (Rana et al., 2022). Rational coordination between the two core indicators underpins accurate pathogen screening, reliable epidemiological supervision and timely epidemic intervention under the One Health strategy.

## 3 Rapid detection technologies for zoonotic pathogens

**Figure 2:**
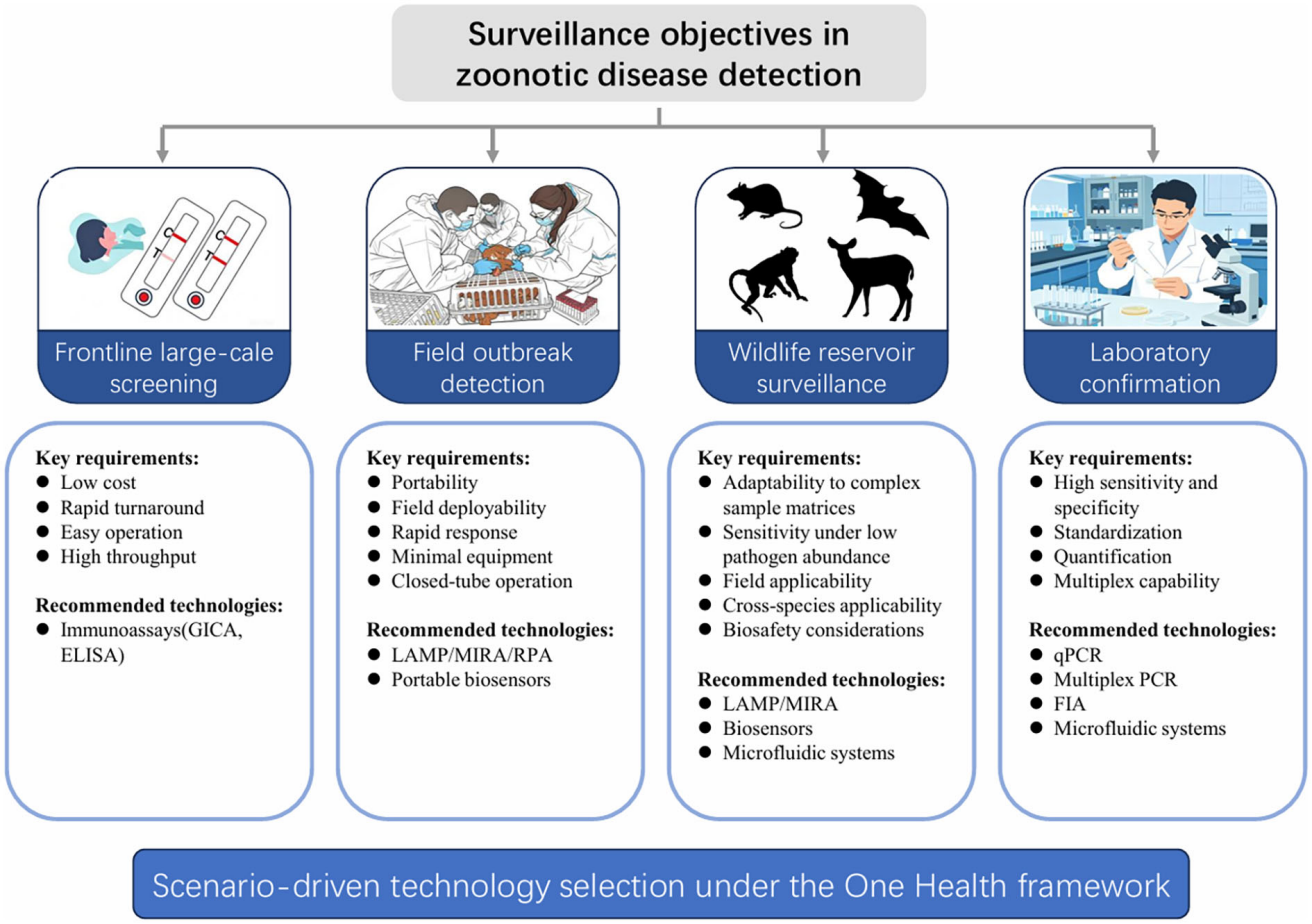
Scenario-driven selection framework for rapid detection technologies in zoonotic disease surveillance. Zoonotic disease detection technologies should be guided by surveillance objectives rather than solely by analytical performance. Frontline and laboratory testing differ in requirements, making no single platform universally suitable for One Health applications.

Given the diverse diagnostic requirements across different zoonotic surveillance scenarios, the selection of appropriate detection technologies should be guided by application context rather than analytical performance alone. To provide a systematic framework for scenario-based technology selection, we summarize the relationships among surveillance objectives, key operational requirements, and suitable diagnostic platforms in **Figure 2**. This framework highlights how different technologies can be optimally deployed across frontline screening, field outbreak detection, wildlife reservoir surveillance, and laboratory confirmation within the One Health context.

### 3.1 Conventional rapid detection technologies

Conventional rapid detection technologies are primarily based on immune-serological methods and rely on the specific interaction between antigens and antibodies, with signal amplification used to achieve rapid pathogen screening (Zeng et al., 2018; Zhang et al., 2024a). Owing to their operational simplicity, low cost, and minimal technical requirements, these approaches remain widely used in primary-level surveillance and frontline disease control, particularly in resource-limited settings (**Table 1**).

**Table 1:** Multi-dimensional comparison of rapid detection technologies for zoonotic pathogens.

Technology type	Detection time	Sensitivity (nucleic acid; bacteria)	Specificity	Equipment dependency	Multiplexing capability	Typical zoonotic surveillance scenaria	Zoonotic detection adaptability	Typical zoonotic pathogen examples	Reference
Colloidal Gold Immunochromatographic Assay (GICA)	5–15 min	10-100 ng/mL 10²-10^4^ CFU/mL	Moderate-High	None	Low	Farm-level screening; primary healthcare triage; slaughterhouse or border checkpoint screening	High for frontline screening; low for early infection and low-load wildlife samples	SARS-CoV-2, E. coli O157: SFTSV, Brucella	(Zuo et al., 2018; Wu et al., 2021; Lu et al., 2024; Wang et al., 2025c; Wu et al., 2025)
Rapid ELISA	60–120 min	~10 ng/mL; ~10³ CFU/mL	Moderate-High	Microplate Reader (Optional)	Moderate	Batch testing in primary veterinary and public health laboratories	Moderate to high for serological screening; limited for immediate field deployment	Toxoplasma gondii, Brucella, Listeria, Salmonella, Influenza A,	(Chen et al., 2008; Cautivo et al., 2014; Wang et al., 2015; Liew et al., 2025; Miao et al., 2025)
Fluorescence Immunoassay (FIA)	15–30 min	~10 pg/mL; 10~100 CFU/mL	High	Fluorescence Detector	Moderate	Mid-level laboratory quantitative antigen/antibody testing	Moderate; useful where rapid quantification is needed but instrument access is available	High-sensitivity quantitative detection of antigens/antibodies: Influenza virus, RSV, SARS-CoV-2, Brucella,	(Chen et al., 2010; Lu et al., 2021; Li et al., 2022; Wang et al., 2025a)
qPCR	60–120 min	10–100 copies/reaction; 10 CFU/g	Very High	qPCR System	Moderate	Reference laboratory confirmation; interspecies surveillance comparison; pathogen load monitoring	Very high for confirmatory diagnosis; limited for remote field deployment	Gold standard for nucleic acid detection: Avian Influenza Virus, Brucella spp., Rabies virus, Anthrax	(Guri et al., 2024; Ochai et al., 2024; Fan et al., 2025b; Meng et al., 2025; Priya et al., 2025)
LAMP	30–60 min	10–100 copies/reaction; 10–100 CFU/mL	Moderate-High	Constant Temperature Bath/Portable Heater	Low	Wildlife-associated field screening; farm outbreak detection; decentralized molecular testing	High	Rapid field nucleic acid detection: Avian influenza, Ebola virus, Leptospira spp., Leishmania, Ehrlichia ruminantium	(Nakao et al., 2010; Kurosaki et al., 2016; Fan et al., 2025a; Glazunova et al., 2025; Hamer et al., 2025; Robène et al., 2025; Xie et al., 2025)
MIRA	15–40 min	10^3^ copies/mL; 10^2^–103 CFU/mL	High	None	Moderate	Emergency field response; rapid molecular confirmation in resource-limited settings	Very high	Novel isothermal amplification for rapid field detection: Viruses (e.g., SARS-CoV-2, Influenza, adenovirus), Bacteria (e.g., Brucella spp., Yersinia pestis).	(Lai et al., 2022; Chang et al., 2024; Dai et al., 2024; Dong et al., 2025; Hu et al., 2025)
RPA	15–30 min	10^3^–10^4^ copies/mL; ~10^3^ CFU/mL	High	Portable Heater	Moderate	Ultra-rapid emergency detection; mobile outbreak investigation	High, but constrained by reagent cost	Ultra-rapid isothermal amplification: Ebola virus, Lassa virus, Bacillus anthracis.	(Ceruti et al., 2024; Chen et al., 2025; Eiamphungporn et al., 2025; Wang et al., 2025d)
Electrochemical Sensor	10–30 min	10^3^ copies/mL; 1–100 CFU/mL	High	Portable Sensor	Moderate	Point-of-care screening at human–animal interfaces	Moderate to high; promising for portable quantitative applications	Emerging POCT technology: Avian Influenza Virus (H5N1, H7N9), Dengue virus, West Nile Virus.	(Kaya et al., 2021; Manring et al., 2022; Yoo et al., 2025; Zhou et al., 2025)
Microfluidic Chip	15–60 min	10^3^ copies/mL; 10–10^4^ CFU/mL	Very High	Dedicated Portable Detector	High	Integrated multi-pathogen screening across One Health surveillance nodes	High, especially for multiplexing and closed-system analysis	Integrated “Lab-on-a-Chip” for multiplex detection: Multiple respiratory pathogens (e.g., SARS-CoV-2, Influenza) or blood-borne/foodborne pathogens.	(Zhao et al., 2019; Gao et al., 2022; Feng et al., 2024; Zhang et al., 2025)

#### 3.1.1 Colloidal gold immunochromatographic assay

The colloidal gold immunochromatographic assay (GICA) is one of the most widely applied rapid diagnostic technologies. Its core principle involves immobilizing specific antigens or antibodies on a nitrocellulose (NC) membrane to form a test line. Colloidal gold–labeled probes (antibodies or antigens) bind to target analytes in the sample and migrate along the NC membrane via capillary action, where accumulation at the test line generates a visible red band that allows qualitative detection by the naked eye (Wang et al., 2022; Lu et al., 2024; Li et al., 2025a).

GICA is characterized by an exceptionally rapid turnaround time, typically producing results within 5–15 minutes, and requires no specialized instruments beyond simple sample application, making it well suited for point-of-care testing (POCT) and on-site use (Law et al., 2014; Li et al., 2025a). Moreover, the extremely low cost of test kits—generally ranging from USD 1 to 5 per assay—and the direct visual interpretation of results without the need for professional training have facilitated its widespread deployment in large-scale screening programs (Li et al., 2011). As a result, GICA has been extensively applied for the rapid screening of pathogens such as avian influenza virus, Brucella spp., rabies virus, and SARS-CoV-2, with antigen test kits for COVID-19 becoming indispensable tools in frontline disease control (Wang et al., 2010; Prakash et al., 2021; Li et al., 2025a). GICA has also been applied for the rapid detection of other important zoonotic pathogens, including Ebola virus (Green, 2022), severe fever with thrombocytopenia syndrome virus (SFTSV) (Zuo et al., 2018), and hantaviruses (de Souza and Figueiredo, 2016). Besides, colloidal gold strip assays targeting H5 and H7 avian influenza viruses have been widely used for rapid poultry farm surveillance and live-bird market screening, enabling timely identification of infected flocks before laboratory confirmation (Sun et al., 2018; Fu et al., 2023). In brucellosis control programs, GICA-based antibody detection kits have been deployed for rapid screening of livestock and occupationally exposed populations in resource-limited endemic regions (Tong et al., 2025). Similarly, rabies virus GICA kits have been developed for rapid antibody detection in animal serum samples, facilitating emergency diagnosis in field veterinary settings (Tao and Li, 2014). During the COVID-19 pandemic, SARS-CoV-2 antigen lateral flow assays represented one of the largest global deployments of GICA technology, supporting mass community screening, border inspection, and household self-testing on an unprecedented scale (Wu et al., 2025).

Despite these advantages, GICA has inherent limitations. Its analytical sensitivity is relatively low, with viral detection limits typically in the range of 10–10^2^ ng/mL (He et al., 2023; Ghorbani et al., 2024), which increases the risk of false-negative results, particularly during early stages of infection. The method provides only qualitative results and cannot differentiate pathogen load. Moreover, specificity may be compromised by cross-reactivity, with false-positive rates exceeding 5% reported for some commercial kits (Xiao et al., 2020; Altamimi et al., 2021). GICA is also poorly suited for multiplex detection, as most assays are designed to detect only a single pathogen per test. In zoonotic disease control, GICA is particularly valuable for decentralized frontline screening, such as farm entry surveillance, slaughterhouse inspection, and rapid triage during outbreaks, although its lower sensitivity limits its utility for early-stage infection and wildlife reservoir surveillance.

#### 3.1.2 Rapid enzyme-linked immunosorbent assay

Rapid indirect and competitive ELISA formats have been increasingly developed and applied for zoonotic pathogen detection in recent years, complementing the commonly used sandwich and blocking ELISA formats (Liew et al., 2025; Miao et al., 2025). Rapid ELISA technologies are derived from conventional ELISA through optimization of assay components and workflows, including the use of high-affinity monoclonal antibodies, enhanced enzymatic signal amplification strategies, and markedly shortened incubation times, which are typically reduced from 3–4 h to 30 min (Tongdee et al., 2020; Singampalli et al., 2022; Miao et al., 2025). Compared with traditional ELISA, these improvements significantly accelerate the detection process while preserving the fundamental advantages of ELISA-based immunoassays, such as high specificity and reliability (Wang et al., 2015; Liew et al., 2025).

Compared with GICA, rapid ELISA offers higher analytical sensitivity, with limits of detection generally reaching 10 ng/mL (Singampalli et al., 2022) In addition, absorbance-based readouts allow semi-quantitative assessment of pathogen or antibody levels (Khelfi, 2024). Owing to its balance between detection performance and operational cost, rapid ELISA is particularly suitable for batch testing in laboratory settings and is therefore widely applied in primary or regional laboratories for large-scale screening programs, including serological detection of brucellosis and antibody surveillance of avian influenza virus (Xiao et al., 2021; Legesse et al., 2023). In rabies surveillance, rapid ELISA assays targeting rabies virus glycoprotein antibodies have been employed to evaluate vaccination efficacy in domestic dogs and wildlife reservoirs (Tao and Li, 2014). Furthermore, rapid ELISA methods have been developed for the serological detection of hantavirus (Cautivo et al., 2014).

Nevertheless, rapid ELISA still depends on basic laboratory infrastructure, such as microplate readers, and therefore cannot achieve complete field portability. The operational procedures are also more complex than those of GICA and generally require trained personnel. Furthermore, although substantially shortened compared with conventional ELISA, the total detection time of rapid ELISA (typically around 1h) remains longer than that of immunochromatographic assays (Li et al., 2011; Tongdee et al., 2020; Singampalli et al., 2022).

#### 3.1.3 Fluorescence immunoassay

Fluorescence immunoassay (FIA) combines antigen–antibody reactions with fluorescence signal detection by labeling antibodies or antigens with fluorescent markers (Hicks, 1984). Quantitative analysis is achieved by correlating fluorescence intensity with target concentration. Representative FIA formats include fluorescence polarization immunoassay (FPIA) and time-resolved fluorescence immunoassay (TRFIA). FPIA is a homogeneous, separation-free format that exploits the rotational diffusion difference between free and antibody-bound fluorophore-labeled tracers, enabling rapid competitive quantification of small-molecule analytes within minutes without washing steps (Smith and Eremin, 2008). TRFIA employs lanthanide chelate labels (e.g., Eu³^+^, Tb³^+^) with long fluorescence lifetimes and large Stokes shifts; by introducing a temporal delay between excitation and signal acquisition, it suppresses background autofluorescence and achieves sensitivities one to three orders of magnitude higher than conventional immunoassays, making it well suited for the ultrasensitive detection of zoonotic pathogen antigens and antibodies in complex matrices (Hagan and Zuchner, 2011).

FIA offers substantially improved analytical performance compared with conventional immunochromatographic assays, with detection limits reach to ~50 fg/mL in SARS-CoV-2 antigen and high specificity (Li et al., 2022). The technique supports quantitative detection across a wide dynamic range while maintaining relatively short assay times, achieving a favorable balance between speed and accuracy. The actual duration varies with platform: lateral flow assay (LFA) formats typically deliver results within 30 min (Sajid et al., 2015; Koczula and Gallotta, 2016; Wang et al., 2025b), whereas plate-based methods generally require 1–4 h depending on the specific protocol (Hagan and Zuchner, 2011). These characteristics make FIA particularly suitable for precise and rapid detection in well-equipped laboratories, such as quantitative analysis of Salmonella and Listeria in food samples or viral load assessment in animal-derived specimens (Liu et al., 2021; Wang et al., 2025b). TRFIA has been successfully used for sensitive detection of Brucella and *Mycobacterium tuberculosis* (Lu et al., 2021; Uddin et al., 2026). It is also capable of rapid detection of H10 avian influenza virus (Wang et al., 2026).

Nevertheless, the broader application of FIA is constrained by its reliance on dedicated fluorescence detection instruments, which are associated with higher equipment costs (approximately USD 1,000–50,000) (Zhang et al., 2022). Besides, fluorescent dyes are susceptible to photobleaching, requiring careful handling and timely processing of fresh samples to ensure reliable results (Campos et al., 2026).

### 3.2 Rapid molecular biology–based detection technologies

Rapid molecular biology–based detection technologies rely on the specific amplification and detection of pathogen nucleic acids. By directly targeting conserved or species-specific genetic fragments, these methods achieve markedly higher sensitivity and specificity than culture-based or immunological assays and currently constitute the dominant approach for laboratory-based rapid diagnosis of zoonotic pathogens (**Table 1**).

#### 3.2.1 Real-time quantitative PCR

Real-time quantitative PCR (qPCR) is an extension of conventional PCR that incorporates fluorescent probes, such as TaqMan probes, or intercalating dyes, such as SYBR Green I, enabling real-time monitoring of fluorescence signal changes during amplification. This approach allows both qualitative identification and accurate quantification of target pathogens. Owing to its extremely high analytical sensitivity, with limits of detection typically ranging from 1 to 100 copies/reaction, and its strong specificity (generally ≥98%) achieved through probe-based assay design, qPCR is widely regarded as the benchmark method for molecular diagnosis (Guri et al., 2024; Fan et al., 2025b; Meng et al., 2025). Moreover, qPCR provides reliable quantitative results across a broad dynamic range spanning 5–7 orders of magnitude, while the total detection time is typically reduced to 1–2 hours compared with conventional PCR (Bastien et al., 2008; Jones et al., 2016).

These performance characteristics make qPCR particularly suitable for laboratory confirmation of infection, pathogen load monitoring, and molecular epidemiological investigations. It has therefore become the “gold standard” rapid diagnostic method for many zoonotic diseases, including nucleic acid detection of SARS-CoV-2 (Dutta et al., 2022), genotyping of Brucella species (Naranchimeg et al., 2025), and detection of anthrax in livestock (Ochai et al., 2024).

However, the widespread application of qPCR is constrained by several factors. The technique requires dedicated real-time PCR instruments, which are costly and limit deployment in resource-limited settings. Moreover, the workflow involves multiple steps, including nucleic acid extraction and amplification, and thus depends on trained personnel. The presence of inhibitory substances in complex biological samples may also interfere with amplification efficiency, necessitating high-quality nucleic acid templates. In this context, qPCR remains especially important for confirmatory diagnosis, pathogen load assessment, inter-host comparison, and surveillance programs that require high analytical confidence across human, animal and environmental samples under the One Health framework (Gebreyes et al., 2014).

#### 3.2.2 Multiplex PCR technologies

Multiplex PCR technologies build upon the basic PCR framework by enabling the simultaneous amplification of multiple pathogen-specific target genes within a single reaction system through the incorporation of multiple sets of primers and probes (Yan, 2015). By detecting several pathogens in parallel, multiplex PCR substantially improves diagnostic efficiency and reduces both sample consumption and per-test costs, making it particularly advantageous for the screening of co-infections or syndromic panels.

In practical applications, multiplex PCR is widely used for the rapid differential diagnosis of mixed infections, such as the combined detection of respiratory pathogens including influenza viruses, respiratory syncytial virus, and adenovirus, as well as the synchronous screening of enteric pathogens such as Salmonella, Shigella, and Escherichia coli (Tan et al., 2023; Serapide et al., 2025).

Despite these advantages, multiplex PCR presents notable technical challenges. Primer and probe design is inherently more complex than in singleplex assays, increasing the risk of primer–dimer formation and nonspecific amplification. What’s more, competitive interactions among targets during multiplex amplification can result in partial inhibition, often leading to a modest reduction in sensitivity compared with single-target PCR assays (Elnifro et al., 2000).

#### 3.2.3 Rapid nucleic acid extraction technologies (supporting technologies)

Nucleic acid extraction represents a critical rate-limiting step in molecular diagnostic workflows. Traditional extraction methods, such as phenol–chloroform extraction, typically require 1–2 hours and involve labor-intensive procedures, which are incompatible with rapid detection demands. In recent years, the development of rapid nucleic acid extraction technologies has significantly improved overall diagnostic turnaround times and enhanced the practicality of molecular assays (Yuan et al., 2022; Wang et al., 2024b).

Among these approaches, magnetic bead–based methods enable rapid nucleic acid extraction within 10–15 minutes through selective adsorption and magnetic separation, while membrane filtration–based methods further simplify workflows, allowing extraction to be completed in as little as 5–10 minutes (Chen et al., 2020; Li et al., 2023). In parallel, direct amplification strategies have emerged that eliminate the need for a standalone extraction step altogether. By optimizing amplification systems to tolerate inhibitory substances present in raw samples, these methods allow direct sample input, further reducing detection time and operational complexity. Together, these supporting technologies play a crucial role in improving the speed, robustness, and field applicability of molecular biology–based rapid detection systems (Wang et al., 2023).

### 3.3 Emerging isothermal amplification technologies

Isothermal amplification technologies enable efficient nucleic acid amplification at a constant temperature, typically between 60 and 65 °C, without the need for thermal cycling instruments (Srivastava and Prasad, 2023). By combining the high analytical sensitivity characteristic of molecular diagnostic methods with the portability of conventional rapid tests, isothermal amplification has emerged as a pivotal technological breakthrough for on-site and field-based pathogen detection (**Table 1**).

#### 3.3.1 Loop-mediated isothermal amplification

Loop-mediated isothermal amplification (LAMP) was first reported by Notomi and colleagues in 2000 (Parida et al., 2008). The method relies on the design of four primers, including forward inner primer (FIP)/backward inner primer (BIP) and forward outer primer (F3)/backward outer primer (B3), targeting six distinct regions of the target gene and exploits the strand displacement activity of Bst DNA polymerase to achieve exponential nucleic acid amplification under isothermal conditions. Amplification products can be directly visualized either by the formation of a white precipitate of magnesium pyrophosphate or through colorimetric or fluorescent dyes, allowing result interpretation without electrophoresis or specialized detection instruments (Parida et al., 2008).

LAMP offers several notable advantages for rapid diagnostics. The assay is highly efficient, with amplification typically completed within 30–60 minutes, and exhibits high analytical sensitivity, with detection limits generally ranging from 1 to 100 copies/μL, and in some optimized systems reaching as low as 1 copy/μL (Fan et al., 2025a; Glazunova et al., 2025). Equipment requirements are minimal, as amplification can be performed using a simple water bath or a portable constant-temperature device. Additionally, LAMP demonstrates strong tolerance to inhibitory substances commonly present in crude nucleic acid extracts, enhancing its robustness in field applications (Soroka et al., 2021; Nwe et al., 2024). Together, these features have supported its practical application across a wide range of zoonotic pathogens in both laboratory and field settings, including Ebola virus (Kurosaki et al., 2016), Zika virus (Silva et al., 2019), *Brucella* spp (Mu et al., 2022), and *Leptospira* spp (Hamer et al., 2025). Besides, LAMP have been validated for rapid detection of monkey malaria (Lai et al., 2024), Leishmania (Nzelu et al., 2019), and Ehrlichia ruminantium (Nakao et al., 2010), a tick-borne zoonotic pathogen of ruminants.

Despite its strengths, LAMP also has inherent limitations. Primer design is relatively complex, requiring 4–6 primers to target 6–8 distinct regions, which prolongs assay development for newly emerging pathogens (Yang et al., 2024). Its specificity, typically in the range of 90–95%, is slightly lower than that of qPCR, and false-positive results may occur (Parida et al., 2008; Silva et al., 2019; Moore et al., 2021). Furthermore, the large quantity of amplification products increases the risk of aerosol contamination when reaction tubes are opened, posing challenges for assay containment. LAMP is also less amenable to multiplexing, with most conventional systems detecting only two to three targets simultaneously, while optimized protocols enable multiplex detection of up to five pathogens (Silva et al., 2019; Moore et al., 2021). Its portability and tolerance to crude samples make LAMP especially attractive for zoonotic field surveillance, including livestock screening and wildlife-associated outbreak investigations.

#### 3.3.2 Multienzyme isothermal rapid amplification

Multienzyme isothermal rapid amplification (MIRA) is based on the endogenous DNA homologous recombination and repair mechanisms found in living organisms. The technique employs a suite of genetically engineered functional proteins—including recombinase (e.g., RecA), DNA helicase, single-stranded DNA-binding proteins, and DNA polymerase—that act synergistically to achieve rapid, exponential nucleic acid amplification under constant, low-temperature conditions ranging from 25 to 42 °C (Park and Zhang, 2024). Amplification can typically be completed within approximately 15 minutes using only two primers. The resulting products can be detected through multiple readout formats, including agarose gel electrophoresis, real-time fluorescence monitoring, or colloidal gold–based lateral flow strips, providing flexibility across laboratory and field settings (Zhu et al., 2024).

MIRA is distinguished by its exceptionally rapid reaction kinetics and high analytical performance. The amplification process is usually completed within 15 minutes while maintaining high sensitivity and strong specificity, with reported specificity levels reaching 97–98% (Park and Zhang, 2024; Zhu et al., 2024). The use of a coordinated multienzyme system, together with carefully designed primers, effectively reduces the risk of nonspecific amplification and false-positive results. The platform also supports universal detection of both RNA and DNA pathogens, as reverse transcriptase can be integrated into the reaction system to enable one-step amplification (Ji et al., 2022). Compared with traditional LAMP assays, MIRA exhibits superior multiplexing capability, allowing the simultaneous detection of three to five targets within a single reaction. In addition, the method is operationally simple, requiring only constant low-temperature conditions without precise thermal cycling equipment, and demonstrates strong tolerance to inhibitors commonly present in crude sample preparations.

Owing to these characteristics, MIRA is well suited for rapid molecular diagnostics across a wide range of application scenarios, including on-site testing, primary-level surveillance, and laboratory-based analysis. It is particularly advantageous in emergency diagnostics and mixed-infection screening, such as combined detection of SARS-CoV-2 and influenza viruses (Yang et al., 2025). It has also been widely applied in on-site identification of zoonotic pathogens, enabling rapid field detection of *Schistosoma japonicum* (Lesheng et al., 2024), and *Mycobacterium tuberculosis* (Chang et al., 2024). Beyond infectious disease surveillance, MIRA has also demonstrated utility in food safety monitoring and genetically modified organism (GMO) detection (Chen et al., 2021). Despite its considerable advantages, MIRA has several limitations. The complexity of the multienzyme system imposes high requirements on manufacturing processes, resulting in reagent costs that are generally higher than those of LAMP-based assays. For certain extreme sample types with high levels of inhibitory substances, additional optimization of sample pretreatment may still be required. Furthermore, maintaining enzyme stability typically necessitates cold-chain storage and transportation, and large-scale technology deployment remains dependent on the wider availability of compatible portable detection devices. MIRA is particularly promising for One Health applications because its rapid reaction speed and low-temperature requirement support flexible deployment across farm, field, and emergency surveillance settings.

#### 3.3.3 Other isothermal amplification technologies

In addition to LAMP and MIRA, several other isothermal amplification methods have been developed and applied in rapid pathogen detection. Recombinase polymerase amplification (RPA) relies on the coordinated action of recombinase, single-stranded DNA-binding proteins, and DNA polymerase to achieve rapid amplification at relatively low temperatures (37–42 °C). The entire amplification process can be completed within 10–20 minutes, with detection limits as low as 10 copies/μL and high analytical specificity, making RPA particularly attractive for ultra-rapid diagnostics (Lobato and O'Sullivan, 2018; Xie et al., 2026). Nevertheless, the relatively high cost of reagents, typically USD 20–30 per assay, remains a major constraint.

Cross-priming amplification (CPA) employs a cross-primer design to form stem–loop structures and achieves amplification at approximately 63 °C within 30 minutes. Compared with LAMP, CPA requires simpler primer design and lower technical barriers, making it a practical option for low-cost, primary-level diagnostic applications, although its overall performance is generally intermediate between LAMP and RPA (Xu et al., 2012; Huang et al., 2024).

### 3.4 Other rapid detection technologies

#### 3.4.1 Biosensor technologies

Biosensor technologies integrate biological recognition elements—such as antigens or antibodies, nucleic acid probes, or enzymes—with physicochemical transducers to convert specific biological interactions into measurable signals, including electrochemical, optical, or piezoelectric outputs (Bhatia et al., 2024). By directly translating biomolecular recognition events into quantitative signals, biosensors enable rapid and sensitive pathogen detection and are widely regarded as a core technological direction for the future development of intelligent and automated diagnostic systems (Castillo-Henríquez et al., 2020).

Among these approaches, electrochemical biosensors detect changes in electrochemical properties—such as current, potential, or impedance—at the electrode surface induced by the binding of pathogens to their recognition elements. These systems typically achieve detection limits in the range of 10–100 copies/μL, with assay times of approximately 5–20 minutes (Ali et al., 2018; Antiochia, 2022). Owing to their compact design and low power consumption, electrochemical biosensors can be miniaturized into handheld devices resembling glucose meters, making them particularly suitable for on-site quantitative testing. Such portable electrochemical platforms have already been applied to the detection of pathogens including SARS-CoV-2 and Salmonella (Kaya et al., 2021; Feng et al., 2023). Optical biosensors, such as surface plasmon resonance (SPR) and quantum dot–based fluorescence sensors, detect pathogens by monitoring changes in optical signals from biomolecular interactions (Boro et al., 2025). They offer high sensitivity and enable real-time, label-free or fluorescence-based analysis. However, their relatively high cost and system complexity currently restrict their application mainly to laboratory environments (Sharma et al., 2021). In laboratory diagnostic practice, SPR optical biosensors are widely adopted for high-throughput serological detection of Leptospira and specific identification of pathogenic Cryptosporidium, providing reliable technical support for retrospective investigation of zoonotic infectious diseases (Kang et al., 2008; Suhailin et al., 2022).

Piezoelectric biosensors detect pathogens through changes in the resonant frequency of a quartz crystal caused by mass shifts upon binding. This label-free approach is simple and does not require signal amplification, but its lower sensitivity compared to other biosensor types makes it more suitable for preliminary screening (Pohanka, 2025).

#### 3.4.2 Microfluidic chip technologies

Microfluidic chip technologies employ microfabrication techniques to construct microchannels, reaction chambers, and integrated functional units on a single chip, enabling the seamless integration of sample pretreatment, nucleic acid extraction, amplification, and detection into a unified platform (Zhao et al., 2019; Gao et al., 2022). These systems require only microliter-scale sample volumes, achieve rapid detection within 15–30 minutes, and substantially reduce the risk of cross-contamination through closed-system operation (Zhao et al., 2019).

Microfluidic platforms excel at portable, high-precision diagnostics, as exemplified by integrated SARS-CoV-2 detection chips and multiplex systems for concurrent pathogen screening (Jamiruddin et al., 2022). They have been successfully deployed to detect key zoonotic pathogens, including Vibrio cholerae (Sayad et al., 2018), Zika virus (Mauk et al., 2017), and *Mycobacterium tuberculosis* (Ismail et al., 2023). In recent years, fully integrated microfluidic devices paired with portable detectors have emerged, streamlining diagnostic workflows into a simple “sample-to-result” process. This progress has promoted the gradual adoption of microfluidic technologies in primary care and field surveillance settings (Wang et al., 2025e).

Despite their promise, microfluidic chip technologies face several challenges that currently limit large-scale adoption (Wang et al., 2025e; Zhang et al., 2025). Chip fabrication costs remain relatively high, and mass production with consistent quality is technically demanding. Particularly, poor cross-platform compatibility persists, as different chip designs often require dedicated, proprietary detection instruments, which constrains standardization and broader dissemination.

## 4 Technology adaptation across major zoonotic surveillance scenarios

### 4.1 Wildlife reservoir surveillance

Wildlife reservoir surveillance often involves low pathogen abundance, intermittent shedding, and complex sample matrices such as feces, oral swabs, carcass tissues, or environmental residues. Under these conditions, highly portable and inhibitor-tolerant methods are particularly valuable. LAMP, MIRA, and selected biosensor-based approaches show strong potential because they can be deployed near sampling sites and reduce the need for centralized laboratory transport (Das et al., 2022; Dong et al., 2025; Robène et al., 2025; Yang et al., 2025). However, where biosafety and confirmatory accuracy are critical, qPCR remains necessary after initial field screening.

### 4.2 Farm-level and veterinary frontline screening

At the farm level, rapid decision-making is essential for outbreak containment, quarantine management, and animal movement control (Doyle and Erickson, 2012; Zhang et al., 2024b). In such scenarios, speed, low cost, and ease of operation are often prioritized over maximal analytical sensitivity. GICA and rapid ELISA are therefore well suited to large-scale frontline screening. Isothermal amplification methods such as LAMP and MIRA provide an important intermediate option by combining relatively high sensitivity with field deployability, especially when rapid molecular confirmation is needed without full laboratory infrastructure.

### 4.3 Emergency outbreak-site detection

During outbreak emergencies, such as suspected avian influenza or hemorrhagic fever events, diagnostic systems must deliver actionable results within a very short timeframe while functioning under constrained conditions. Here, ultra-rapid isothermal amplification platforms, lateral flow-assisted readouts, and portable biosensors are especially useful (De Felice et al., 2022; Lei and Xu, 2024; Dong et al., 2025). Their practical value lies not only in speed, but also in minimizing sample transport delays and enabling immediate local response. Nevertheless, confirmatory laboratory testing remains necessary for epidemiological verification and downstream molecular characterization.

### 4.4 Human–animal interface and One Health integrated surveillance

In integrated surveillance systems under the One Health framework—linking hospitals, veterinary centers, food inspection units, and environmental monitoring programs—comparability and standardization grow increasingly critical, especially at the human–animal interface where zoonotic pathogens frequently emerge and spread (Ghai et al., 2022; Hassan et al., 2023; Cheng et al., 2024). These systems serve as a vital line of defense against cross-species disease transmission, making unified protocols for sample handling, testing procedures, and data formatting essential to avoid fragmented risk assessments. qPCR and multiplex PCR remain central to these networks: their high analytical confidence ensures reliable detection of low-abundance pathogens (a common challenge at the human-animal-environment nexus), while their capacity for pathogen typing or co-detection supports precise tracing of disease origins—from livestock farms to clinical settings (Dale et al., 2016; Hao et al., 2025; Huels et al., 2026). Moreover, these technologies facilitate cross-sector data harmonization, enabling seamless collaboration between public health officials, veterinarians, and food safety experts (Tran et al., 2025). To further strengthen One Health surveillance, microfluidic systems and intelligent biosensor platforms offer impactful enhancements: they enable automated, on-site processing (ideal for remote farms or border inspection points), generate real-time digital reports to eliminate manual errors, and support distributed data sharing via cloud-based platforms—ensuring timely alerts about emerging threats at the human–animal interface (Gao et al., 2022; Feng et al., 2024; Wang et al., 2025e; Liu et al., 2026).

Taken together, no single rapid detection technology is optimal for all zoonotic disease applications. Rather, diagnostic selection should be guided by surveillance objective, host type, sample matrix, infrastructure availability, biosafety requirements, and whether the task involves initial screening, emergency triage, or confirmatory diagnosis. This scenario-oriented perspective is particularly important for zoonotic diseases because their detection spans multiple hosts and operational environments under the One Health framework.

## 5 Current bottlenecks and unmet needs

Sample pretreatment remains a critical bottleneck affecting diagnostic accuracy in rapid detection systems. Complex biological matrices—such as blood, feces, and tissue homogenates—contain inhibitory substances including proteins, polysaccharides, and nucleases that can interfere with nucleic acid amplification and signal readout (Buckwalter et al., 2014; Sidstedt et al., 2020; Nwe et al., 2024). Although rapid extraction techniques have been developed to address this, many diagnostic platforms still rely on relatively purified inputs. Moreover, existing methods often fall short under field conditions due to issues with cost, reagent stability, and operational complexity (Özay and McCalla, 2021). These limitations are especially pronounced in resource-limited settings where laboratory infrastructure is minimal or absent (**Figure 1**).

Another persistent challenge is the lack of standardization and quality control across diagnostic platforms and manufacturers. Substantial performance variability has been reported among commercial kits—even when using the same detection principles—particularly for immunochromatographic assays. Sensitivity differences of several folds have been observed, often linked to inconsistencies in antibody quality, reagent formulation, or manufacturing precision (Olive et al., 2020; van der Wal et al., 2025). The absence of harmonized technical standards and external quality assessment programs hampers inter-study comparability and weakens surveillance systems through data fragmentation and reduced interoperability across regions.

Furthermore, accessibility of advanced diagnostic platforms at the grassroots level remains insufficient. Cutting-edge methods—such as multienzyme isothermal amplification and biosensor-integrated detection systems—often rely on expensive enzymes, temperature-controlled reagents, or proprietary instruments that restrict large-scale deployment in developing regions and remote field settings. Additionally, assay complexity increases the likelihood of operator errors, particularly when administered by personnel with limited technical training. Finally, the capacity for rapid adaptation to newly emerging or genetically evolving zoonotic pathogens remains limited. The development timeline for new assays often spans several months, delaying early identification and containment efforts during outbreaks (Yager et al., 2008; Yamin et al., 2023). This highlights the urgent need for agile and modular diagnostic platforms that support rapid reconfiguration in response to novel threats.

## 6 Future perspectives under the One Health framework

To address these challenges, current research and development efforts in rapid detection technologies are increasingly converging along several trajectories. One prominent trend is the move toward highly integrated and automated diagnostic systems that combine sample pretreatment, nucleic acid amplification, signal detection, and result interpretation within a single platform. Such “sample−in, answer−out” solutions, exemplified by integrated microfluidic chip systems and portable all−in−one analyzers, have the potential to greatly simplify workflows while minimizing contamination risk and operator dependence (Wang et al., 2024a).

In this context, multienzyme isothermal rapid amplification (MIRA) stands out as a particularly promising core technology for next−generation integrated systems. Its fast reaction kinetics (typically within 15 minutes), high specificity, intrinsic contamination control (e.g., UNG enzyme), and multiplexing capability (3–5 targets per reaction) make it suitable for building compact, automated, and field-deployable molecular diagnostic devices (Dong et al., 2025). Efforts are underway to stabilize enzyme cocktails, reduce costs through improved production, and embed MIRA into modular, cartridge−based systems (Feng et al., 2023). MIRA also enables efficient high-dimensional multiplex detection without the need for thermal cycling, making it ideal for outbreak surveillance. The integration of CRISPR/Cas-based diagnostics, such as SHERLOCK and DETECTR, with MIRA further extends the potential of multiplexed, sensitive, and specific detection. These systems can identify multiple genetic targets within a single reaction, and when combined with barcoding and signal decoding, could support comprehensive, single-assay screening for multiple zoonotic pathogens (Dong et al., 2025; Hu et al., 2025).

Digitalization and intelligent analysis will further reshape rapid diagnostics. Smartphone integration, wireless communication, and IoT-enabled data sharing can provide automated result interpretation and real-time reporting to surveillance networks (Ait Lahcen et al., 2026). MIRA-based platforms, which support fluorescence, lateral flow, and electrochemical readouts, are well-suited for such interfaces, allowing results to be captured and shared remotely—vital for early outbreak warnings in remote settings (Mohammad et al., 2022). Equally critical is cost reduction and equitable access. Advances in enzyme production, local reagent sourcing, and low-cost cartridge materials are expected to reduce per-test costs, improving scalability in low- and middle-income countries. MIRA’s open system and simplified primer design (only two primers needed) make it amenable to local customization and rapid prototyping—an asset during emerging outbreaks (Feng et al., 2023).

Finally, the convergence of molecular and immunological platforms is anticipated to yield hybrid diagnostic systems. These would combine rapid antigen screening with confirmatory nucleic acid detection in a single portable device. In this model, MIRA could serve as the molecular engine, enabling accurate, multiplex confirmatory testing that closes the gap between field screening and laboratory-grade diagnostics (Realegeno et al., 2021; Chen and Meng, 2025; Dong et al., 2025).

## 7 Conclusion

Rapid detection technologies have become indispensable tools for the surveillance and control of zoonotic diseases. However, their value in this field cannot be judged solely by analytical sensitivity or detection speed. Unlike conventional diagnostics for single-host infectious diseases, zoonotic disease detection must address cross-species transmission, diverse sample matrices, wildlife reservoir surveillance, decentralized field deployment, and the broader requirements of One Health monitoring systems. These factors fundamentally shape technology suitability in real-world applications. From this perspective, conventional immunological assays remain useful for large-scale frontline screening, qPCR continues to serve as the reference method for confirmatory diagnosis and intersectoral standardization, while isothermal amplification platforms such as LAMP and MIRA provide particularly strong advantages for field-based and resource-limited zoonotic surveillance. Emerging biosensor and microfluidic technologies further expand the possibilities for integrated, automated, and multiplexed detection. Nonetheless, major challenges remain, including complex sample pretreatment, insufficient standardization across platforms, limited accessibility in remote settings, and the need for rapid adaptation to newly emerging zoonotic threats.

Future progress will depend on the development of diagnostic systems that are not only faster and more sensitive, but also more adaptable to One Health surveillance scenarios. Integrated sample-to-answer platforms, closed-tube field-deployable molecular assays, intelligent digital reporting systems, and cost-reduced multiplex devices will be especially important for strengthening early warning and coordinated response. Ultimately, scenario-driven technology selection and One Health-oriented diagnostic innovation will be essential for improving global preparedness against zoonotic diseases.
